# Faculty clinical articles: a regular update for the oral healthcare team

**DOI:** 10.1038/s41415-022-4445-x

**Published:** 2022-07-08

**Authors:** Martyn T. Cobourne, Stephen R. Porter, Michael Escudier, Matthew Garrett

**Affiliations:** 41415134070001 Professor of Orthodontics, Faculty of Dentistry, Oral and Craniofacial Sciences, King´s College London, London, UK; 41415134070002 UCL, Eastman Dental Institute, UCL Rockefeller Building, 21 University Street, London, UK; 41415134070003 Professor of Oral Medicine, Faculty of Dentistry, Oral and Craniofacial Sciences, King´s College London, London, UK; 41415134070004 Consultant in Restorative Dentistry, Royal National ENT and Eastman Hospitals, University College Hospital NHS Foundation Trust, London, UK

## Abstract

The Faculty of Dental Surgery of the Royal College of Surgeons of England and *British Dental Journal* have teamed up to provide a regular series of short articles on different aspects of clinical and academic dentistry. This series will provide concise insight into a diverse range of topics with the aim of providing regular ongoing professional development for all members of the oral healthcare team. We begin here, with a short update on the Faculty and overview of the series' aims.

## Key points

- The Faculty of Dental Surgery at the Royal College of Surgeons of England and *British Dental Journal *have teamed up to provide a regular series of short articles on different aspects of clinical and academic dentistry.
- Aims to provide insight into a diverse range of topics commissioned from a wide spectrum of authors in an accessible and succinct format.
- This initiative coincides with a new era for the FDSRCS and Royal College of Surgeons of England as the faculty moves into a new building, and combined with the *British Dental Journal*, it is hoped that this series will be enjoyed by all members of the oral healthcare team.

## Introduction

**Fig. 1:**
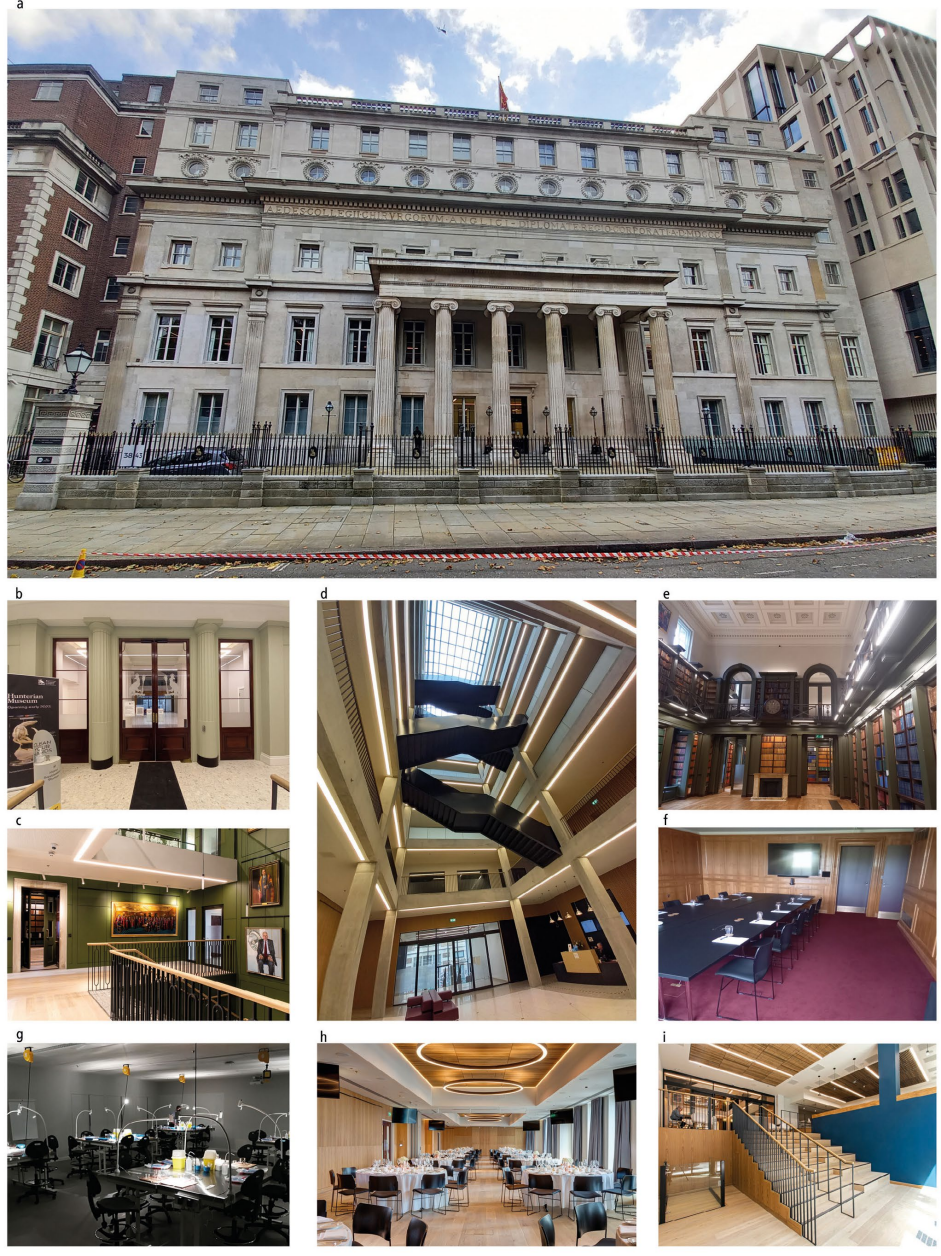
a, b, c, d, e, f, g, h, i) The newly refurbished Royal College of Surgeons of England building in Lincoln's Inn Fields, Central London. The frontage of the original building has been retained, while the front and rear entrances have been redesigned. The interior space is dominated by a light and expansive atrium traversed by the main staircase, the original library has been restored and refurbished, and there are numerous state-of-the-art teaching areas, meeting rooms and conference facilities. The Hunterian museum is currently being redeveloped and is due to open in early 2023

The Faculty of Dental Surgery of the Royal College of Surgeons of England (FDSRCS) is an independent professional body in the UK, committed to enabling dental surgeons and allied dental healthcare professionals to achieve and maintain excellence in their clinical practice and the ongoing care that they provide.^1^ The Faculty was established in 1947 and is an integral component of the Royal College of Surgeons of England located within the main College headquarters in Lincoln's Inn Fields, Central London. The College has stood on this site for over 200 years and recently completed an extensive redevelopment of its headquarters, which has been transformed into a modern, light and flexible main building designed to provide the best education, training and examination facilities, while maintaining the long and prestigious heritage of the original building (Fig. 1).

There are currently around 5,000 members and fellows within the FDSRCS in the UK and internationally. The governing body of the Faculty is the Board,^2^ comprising of 27 members; of which, eighteen are directly elected by members and fellows and nine are invited members, representing key stakeholders in dental education and training. The current Dean is Mr Matthew Garrett who took office in 2020 and will serve one three-year term. The Faculty runs an extensive range of UK and international examinations for dentists and allied dental healthcare professionals for every stage of their career,^3^ which includes Diplomas in Dental Hygiene and Therapy, Orthodontic Therapy and Implant Dentistry; Membership of the Faculty of Dental Surgery; Bi- and Tri-Collegiate Speciality Membership Examinations; and the Fellowship in Dental Surgery by Assessment. The Faculty is also a leading provider of dental professional education, training and career development, both at home and overseas.^4^ It provides a wealth of theoretical and practical support to help all members of the dental team in developing their knowledge and skills through a wide portfolio of accessible platforms and modes of delivery. These include access to multiple journals, including the *Faculty Dental Journal,* *College Bulletin* and* Annals of the Royal College of Surgeons,* as well as e-Den, an online interactive learning resource, delivered in partnership with Health Education England. In addition, the Faculty organises regular masterclasses in areas ranging from special care dentistry to orthodontic wire bending, webinars, careers conferences and revision courses for College examinations. The Faculty has a strong commitment to supporting oral and dental research through a range of research grants and fellowships funded through the College and in partnership with external organisations,^5^ principally the many specialist societies allied to oral healthcare provision and including the British Orthodontic Society, British and Irish Society of Oral Medicine, British Association of Oral Surgeons and British Society of Paediatric Dentistry.

## Life-long learning

A commitment to continuing professional development (CPD) and life-long learning is a key component of being a practising member of the oral healthcare team. In a world of perpetual change and almost continual new advances, it can be challenging to keep up with the changing face of clinical dentistry. Moreover, the COVID-19 pandemic has had a significant influence on many aspects of clinical delivery, education and training in oral healthcare. As part of this remit, FDSRCS and *British Dental Journal* have teamed up to provide a regular series of short articles on different aspects of clinical and academic dentistry that have been carefully selected to be contemporaneous and of interest to the whole oral healthcare team. Collectively, these articles will provide ongoing insight into a diverse range of topics commissioned from a wide spectrum of authors, with the aim of providing regular ongoing CPD in an accessible and succinct format. The subject areas will encompass different aspects of clinical and academic dentistry to include general dental practice, paediatric dentistry, orthodontics, restorative dentistry, periodontics, endodontics, oral surgery, oral medicine and pathology, special care dentistry, radiology, dental public health and the basic sciences relevant to oral health and disease. The articles will incorporate new findings of relevance to oral healthcare management and will be accompanied by clinical examples and a short bibliography. It is hoped that this series will stimulate dentists and allied dental healthcare professionals and provide knowledge of contemporary clinical issues, new advances and ideas in a succinct and readable manner.

This initiative coincides with a new era for the FDSRCS and Royal College of Surgeons of England and combined with the *British Dental Journal*, we hope that this series will be enjoyed by all members of the oral healthcare team.

## References

[1] Royal College of Surgeons of England. Faculty of Dental Surgery. Available at https://www.rcseng.ac.uk/dental-faculties/fds/ (accessed June 2022).

[2] Royal College of Surgeons of England. FDS Board. Available at https://www.rcseng.ac.uk/dental-faculties/fds/faculty/fds-board/ (accessed June 2022).

[3] Royal College of Surgeons of England. Search: Exams. Available at https://www.rcseng.ac.uk/education-and-exams/exams/dental/#Surgical=False&Dental=True (accessed June 2022).

[4] Royal College of Surgeons of England. Search: Courses. Available at https://www.rcseng.ac.uk/education-and-exams/courses/dental/#Surgical=False&Dental=True (accessed June 2022).

[5] Royal College of Surgeons of England. Oral and Dental Research. Available at https://www.rcseng.ac.uk/dental-faculties/fds/research/ (accessed June 2022).

